# Identification and Clinical Significance of Heart Murmurs in Puppies Involved in Puppy Trade

**DOI:** 10.3390/vetsci8080139

**Published:** 2021-07-21

**Authors:** Michela Pugliese, Vito Biondi, Rocky La Maestra, Annamaria Passantino

**Affiliations:** Department of Veterinary Sciences, University of Messina, Via Umberto Palatucci, 98168 Messina, Italy; v.biondi@hotmail.it (V.B.); rlamaestra@unime.it (R.L.M.); passanna@unime.it (A.P.)

**Keywords:** cardiac murmur, puppy, purchase, heart defect

## Abstract

The detection of a congenital heart defect at purchase is an important step in early detection from a clinical and legal standpoint. Indeed, some cardiac abnormalities may be corrected with surgery, and very often, treatment needs to be performed early before congestive heart failure or irreversible heart damage can occur. From a legal viewpoint, if the defect is revealed in a newly purchased puppy, the buyer may be required to return it and receive compensation. Puppies affected with congenital heart defects are likely to die prematurely, causing emotional suffering to the owner. Furthermore, by considering breed predisposition, early recognition allows breeders to avoid breeding from particular dogs with genetic defects and prevent the continuation of genetic defects in breeding lines. Given gaps in the literature about the recognition of murmurs in the puppy trade, the present article describes how to identify a heart murmur in a puppy during a pre-purchase examination and its significance from a clinical and legal viewpoint. In the canine population, the prevalence of cardiac defects ranges between 0.13 and 1.6%. Pulmonic stenosis is the most common defect found in puppies, followed by patent ductus arteriosus, subaortic stenosis, and ventricular septal defect. On the basis of the above considerations, the veterinarian should recognize and identify the murmur following a protocol for routine examination of puppies involved in trade.

## 1. Introduction

Buying a puppy is an important step for most pet owners. The prospective owner aspires for the puppy to be healthy and fit well with the new family, often acting on impulse driven by the opportunity and enthusiasm to get a dog. Instead, a pre-purchase examination before selling is strongly recommended in order to evaluate the health status of the animal. The ground rules for a pre-purchase examination are the same as those applicable to clinical evaluation. The cornerstone is to establish the health of the animal or a specific diagnosis which must then be contextualized in terms of a prospective purchaser’s requirements. On the one hand, there are some buyers for whom any risk is acceptable as they do not perceive clinical signs as a problem, but rather as a normal breed-specific characteristic [1]. On the other hand, some buyers may want assistance to arrive at an informed judgment on the risks related to buying a dog, given that there may be diseases associated with some breeds which reduce the life span or make it unsuitable for its intended use by the purchaser.

Indeed, heart diseases of proven or suspected hereditary origin in puppies may constitute a defect (vice) in buying and selling, making the animal inappropriate for its intended use, or significantly decreasing its value [2]. In cases of congenital and/or hereditary cardiovascular abnormalities that are manifested after the expiration of the vice-free guarantee agreement, conflicts and civil suits might arise [3] between the concerned parties.

Early identification, therefore, assumes great importance, not only to apply a suitable medical and/or surgical workup and formulate a correct prognosis, but also to prevent any litigation.

The diagnostic approach to the discovery of cardiac abnormalities during the auscultation differs in relation to the abnormality detected, the other clinical findings, and the client’s wishes. The innocent murmur is a common physiological finding in puppies, generally as a result of physiologic anemia. Rising hematocrit in growing puppies can explain the spontaneous disappearance of innocent murmurs with aging [4].

Generally, puppies are sold when they are at least 6–8 weeks old to ensure that the puppy has received an adequate supply of milk from the mother dog and has completed the period of socialization.

The discovery of an apparently innocent murmur (hearable discontinuously, early systolic with an intensity of I–II/VI, with the point of maximum intensity in left cardiac base) in a healthy puppy necessitates no more than a follow-up examination at the next appointment for vaccination. Conversely, the detection of a continuous murmur in a puppy requires a complete cardiologic and medical workup.

Once the presence of a murmur has been identified, the aim for the veterinarian during the pre-purchase (clinical) examination is to determine the existence, severity, and exact origin of the underlying cardiac disease. Furthermore, the veterinarian should be required to perform the necessary diagnostic procedures such as echocardiography, which is considered the initial diagnostic test of choice and gold standard for identifying the cause of a cardiac murmur [4], and/or other tests (thoracic radiography, electrocardiography, etc.).

Cardiac examination should be performed to evaluate heart rate, rhythm, and the presence of normal and abnormal sounds. Auscultation should be executed in a silent room, with the animal in standing position, auscultating the precordial valve areas in both sides of the chest and, if an abnormal sound is detected, the point of maximum intensity should be identified.

Given the gaps in the literature regarding a clinical protocol or guidelines for the identification of a cardiac murmur during puppy trade, the authors describe how to categorize heart murmurs in the animal during a pre-purchase examination and its significance from a clinical and legal viewpoint, as murmurs can be physiological or pathological.

## 2. Clinical Examination of the Cardiovascular System

**Figure 1:**
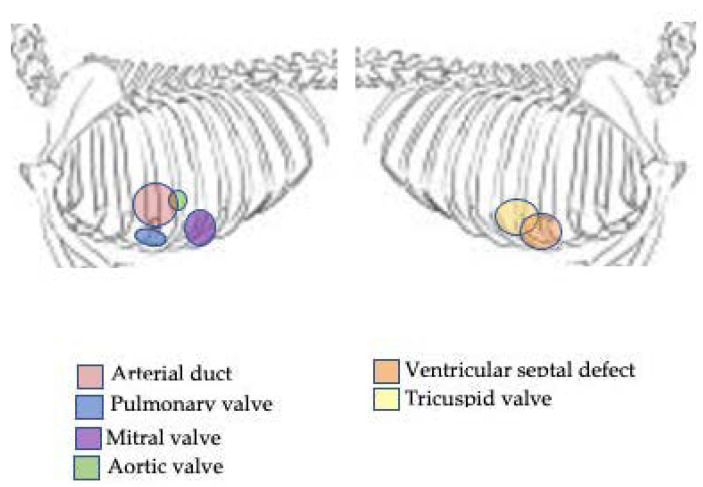
Different sites of auscultation originating the murmur in dog.

Clinical examination is a great help to the veterinarian in the evaluation of the cardiovascular system. Variations in mucous membrane colour and capillary refill time, jugular distension, dependent oedema, tachycardia, and tachypnoea are common findings of congestive heart failure (CHF) [5]. The auscultation of the heart is fundamental for the evaluation of the cardiovascular system. Normally, it is possible to hear two heart sounds: S1, caused by the closing of the atrioventricular valves at the beginning of the systole, and S2, generated by the closing of the semilunar valves at the end of the systolic event. Two other heart sounds also can be found in pathological conditions: S3, caused by the rapid filling of the ventricles in diastole, and S4, generated by atrial contraction. Additional sounds generated by turbulent blood flows are called murmurs. The heart murmurs are classified according to intensity, frequency, quality, duration, configuration, primary location (point of maximum intensity, PMI), and site(s) of radiation [6]. The intensity indicates the loudness of the cardiac murmur and is classified on a scale from 1 to 6 [6], as summarized in Table 1 [6]. The frequency or pitch is an expression of the number of sound waves/second and may be determined with the diaphragm of a stethoscope. This is strongly related to the blood velocity at the site of origin of the murmur and is classified as high, medium, or low. The murmurs that originate from mitral valve insufficiency typically have a high pitch [7], while the murmurs originating from aortic stenosis typically have a low or medium pitch [4,8,9,10]. The quality (or timbre) of the heart murmurs indicates the type of sound, which can be soft, harsh, rumbling, musical or a mixture of these. The shape, also referred to as configuration, indicates the intensity that it assumes during the cardiac cycle; therefore, a murmur may be crescendo, decrescendo, crescendo-decrescendo or plateau. A crescendo murmur gradually increases in intensity, while a *decrescendo* murmur gradually decreases in intensity. A crescendo—decrescendo murmur is characterized by a gradual increase in intensity, followed by a gradual diminution. A plateau murmur conserves a moderate continuous intensity regardless of their intensity grade. The duration (or timing) indicates the portion of the cardiac cycle that the murmur occupies and not in which the murmur onsets. Murmurs may be systolic (early systolic, mid-systolic, late systolic, or holosystolic), diastolic (early diastolic, mid-diastolic, late diastolic, or holo-diastolic) or continuous. A continuous murmur initiates during the systole and continues extending on the second sound, throughout diastole or part of thereof. The location indicates areas on the precordium where the murmur is perceived with a higher intensity and correspond at the site of murmur origin (Figure 1). When a murmur is heard at a site other than the point of maximal intensity, it is said to radiate. The murmur may radiate near to the site of origin following the direction of turbulent jet. An aortic stenosis murmur radiates up the carotid arteries, while mitral murmurs radiate dorsally within the thorax. The first and often most challenging step is to determine the clinical importance of a murmur. It is essential to distinguish pathological heart murmurs from functional ones and, in puppies, from innocent heart murmurs [11].

### 2.1. Innocent Heart Murmur

An innocent heart murmur is found on average in 28% of puppies under the age of 6 months, while in athletic dog breeds such as whippets, it has even higher percentages of up to 58% of puppies [11,12]. It is a systolic heart murmur characterized by a short duration, left PMI, mild degree (I–II/VI), clear character, sometimes defined as a “whistle” or “musical” sound. Innocent murmurs may be dynamic and vary in intensity, based on the level of activity or excitement. The main factor in the formation of the innocent heart murmur is a higher cardiac output in puppies [5]. Furthermore, often, puppies with innocent cardiac murmurs also have a mild physiological anemia which lowers the viscosity of the blood, contributing to the creation of turbulent blood flows [11]. The innocent murmur usually disappears by 6 months of age [5].

### 2.2. Functional Heart Murmur

Also called a physiological murmur, a functional heart murmur is caused by an altered viscosity of the blood or by conditions that increase cardiac output, such as: anemia, fever, pregnancy, hyperthyroidism, or increased sympathetic tone. The functional heart murmur has similar characteristics to the innocent murmur but can also occur in adult dogs. It is generally systolic, short, with mild intensity (I–II/VI grade) and PMI focused on the base of the heart [4].

### 2.3. Pathological Heart Murmur

Heart murmurs of intensities greater than III or VI and PMI on the right side, or associated with arrhythmias or clinical symptoms, should be considered as pathological heart murmurs and require further diagnostic investigations [4].

## 3. Common Congenital Heart Abnormalities

In the canine population, the prevalence of congenital heart disease (CHD) ranges between 0.13 and 1.6% [13]. Single cardiac defects are present in 88.14% of dogs with CHD, while multiple defects are found in the remaining 11.86% of cases [14]. Pulmonic stenosis (PS) is the most common defect found in puppies, followed by patent ductus arteriosus (PDA), subaortic stenosis (SAS), and ventricular septal defect (VSD) [15,16]. Some dog breeds also show a different incidence of some CHDs [17,18]. A complex defect, or combinations of multiple malformations in the same dog, are possible occurrences [19,20].

### 3.1. Pulmonary Stenosis

Pulmonary stenosis (PS) is the most commonly encountered CHD, being described in 11–34% of the total CHD cases in different epidemiological studies [14,15,16,21,22]. Some breeds, such as bulldogs and boxers, show a higher prevalence [14,18,23,24,25]. The disease is classified according to the anatomical location of the narrowing into subvalvular stenosis (right ventricle outflow tract), valvular (pulmonic valve), and supravalvular (main pulmonary artery). Valvular pulmonary stenosis is the most frequent and is classified into two forms:

- Type 1: Dysplasia only affects the valve leaflets, which are fused together but have some mobility, showing a systolic dooming on echocardiographic examination. The aortic/pulmonary trunk diameter ratio is, however, maintained [26].
- Type 2: In addition to dysplasia of the pulmonary valve leaflets as in type 1, there is a hypoplasia of the valve annulus with a diameter ratio between the aorta and the pulmonary trunk greater than 1.5 [26].

The heart murmur of a pulmonary stenosis is holosystolic, with rough timbre, with PMI on the pulmonary valve focus (third intercostal space), poorly or non-radiated, with a “crescendo-decrescendo” pattern. The intensity of the murmur may vary according to the degree of the pathology [27,28] (Table 2).

### 3.2. Patent Ductus Arteriosus

Patent ductus arterial (PDA) is considered the second largest CHD in dogs, with a rate of prevalence between 8 and 26% [13,14,15]. Reverse PDA (rPDA) is much rarer [14]. A predisposition is hypothesized for some dog breeds such as the poodle, German shepherd, pomeranian, Shetland sheep dog, collie, Maltese, Yorkshire terrier, chihuahua and Dutch Stabyhoun [29,30]. During the foetal period, the ductus arteriosus diverts almost 80% of the blood from the pulmonary artery blood bypassing the non-functional lungs [31]. In healthy puppies, the foetal ductus closes within the first days of life, while in puppies affected with PDA, a hypoplasia of the smooth muscle fibres of the vessel prevents the closure, causing its persistence [32]. The size of the ductal opening and the pressure gradient between the pulmonary and systemic circulation influences the amount of blood shunted across the PDA and its direction. The left-to-right shunt is the most common and involves an enlargement of the structures involved in the overflow of blood (left atrium and ventricle, pulmonary trunk, and aorta) leading to left heart failure. In rare cases, if the aberrant duct is very large, the pressure gradient between the pulmonary and systemic circulation can reach an equilibrium. In fact, the pulmonary arteriole can undergo profound modifications, such as remodelling of the muscle component, hypertrophy, and fibrosis, which causes non-reversible pulmonary hypertension [33]. This condition causes a reversal of the shunt, called rPDA, and leads to Einsenmenger Syndrome [34]. The heart murmur of a PDA-affected puppy with left-to-right shunt reveals the pathognomonic continuous murmur, also called Gibson’s murmur, located in the left axillary region. Generally, the murmur has an intensity between the IV/VI and VI/VI degree. The mild increase in aortic systolic pressure and the increase in diastolic pressure determined by the continued diastolic shunting to the pulmonary circulation are the cause of the hyperkinetic femoral pulse [35]. More rarely, an inversion of the shunt to a right-to-left shunt (reverse PDA) may present itself [35,36]. In reverse PDA, there is a marked increase in pulmonary pressure which reverses the shunt, which causes hypertrophy of the right ventricle, and a much less intense murmur. Puppies with an rPDA do not have a murmur, because there is laminar right-to-left or bidirectional ductal flow [36] (Table 2). Quickly identifying the disease is very useful, as surgical repair is contraindicated in conditions of severe pulmonary hypertension, which could also develop over time in puppies with left-to-right PDAs [37,38].

### 3.3. Subaortic Stenosis and Aortic Stenosis

Subaortic stenosis (SAS) and aortic stenosis (AS) have a prevalence between 15 and 21% and 5 and 6% of all CHDs in dogs, respectively [14,15,16]. SAS is the most common CHD of large-breed dogs, such as the rottweiler, golden retriever and boxer [14]. It is defined as a narrowing of the right ventricular outflow tract caused by an abnormal thickening of the endocardial tissue. SAS is classified according to three grades [39]:

- First degree: Small subvalvular endocardial nodules of a few millimetres forming a limited narrowing. There may be no heart murmur at this stage.
- Second degree: The endocardial thickening increases, there is a narrow fibrotic ridge that extends around the left ventricular outflow tract.
- Third degree: Subvalvular obstruction becomes a fibromuscular tunnel involving a large portion of the left ventricle outflow tract and also the mitral valve apparatus.

The AS instead presents obstructive lesions to the aortic valvular apparatus; these may include: hypoplastic aortic annulus or thickened leaflets. Similarly, to SAS, AS also causes left ventricle concentric hypertrophy, and post stenotic dilatation of the aorta and aortic valve insufficiency may also be present [20,40]. The heart murmur of SAS or AS is holosystolic, of the “crescendo-decrescendo” type, with a rough timbre, located at the focus of the aortic valve (fourth intercostal space) but can irradiate. The intensity of the murmur varies according to the severity, it can be very mild up to very strong, with precordial thrill and irradiation towards the base of the heart [19] (Table 2).

### 3.4. Ventricular Septal Defect and Atrial Septal Defect

Ventricular septal defect (VSD) is the fourth most common CHD in the canine population, with an incidence between 5 and 14% [14,15,16], while the atrial septal defect (ASD) makes up about 1–2% of CHDs in total [14,15,16]. For ASD, an inherited basis has been supposed, although never verified [20]. A breed predisposition for boxers, dobermans, old English sheep dogs and Samoyeds has been reported [14,15,16,29].

During embryonic development, the atria and the cardiac ventricles communicate with each other, and only later does the interventricular and atrial septa develop, dividing the heart into four chambers. More specifically, the embryologic development of the atrial septum follows the growth of two atrial membranes [41]. The “primum septum” derives from the roof the atria and goes towards the floor of the atria. The “*secundum septum*” originates from the roof of the atria on the right side of the primum septum. Based on the localization, ASDs are then classified in defects of “primum septum” or “secundum septum”. A defect in the development of the “primum septum” results in an ostium low in the atria, below the foramen ovale onsets for a defect localized near the atrioventricular valves. An anomalous development of the septum secundum determines an ostium at or above the level of the foramen ovale [42]. Similarly, the two ventricles are also separated from each other by septa that join together, forming the interventricular septum. If this does not happen, a communication between the two ventricles remains [20,43]. The most common type of VSD in puppies is located in the basilar perimembranous region, between the aortic flaps and the septal leaflet of the tricuspid valve. Often, the defect alters the geometry and stability of the aortic valvular apparatus, causing valvular insufficiency [14]. As with the PDA, the height and direction of the shunt depends on the size of the communication and the pulmonary and systemic vascular resistances [43]. The shunt is generally left to right and causes an overflow of blood into the left ventricle, pulmonary artery, pulmonary veins, and left atrium. A large VSD can offer little resistance to the passage of blood, and therefore, inevitably generates pulmonary hypertension and reversal of the shunt, which therefore leads to the development of Eisenmenger’s syndrome. Puppies with VSD have harsh, holosystolic heart murmurs, with PMI at the base of the heart with possible diastolic murmur due to aortic insufficiency, and the intensity is related to the severity of the disease. The intensity is related to the severity of the disease and the pressure gradient between left and right ventricles. Given a lower pressure gradient and velocity of shunted blood, the murmur intensity in a dog with a large VSD and some degree of pulmonary hypertension is soft to the auscultation, while the femoral pulse is normal. In dogs affected with moderate ASDs, a grade II to III/VI left basilar holosystolic murmur created by the functional PS volume is hearable. A split S2 heart sound may be present [15] (Table 2).

### 3.5. Tricuspid Valve Dysplasia and Mitral Valve Dysplasia

Tricuspid valve dysplasia (TVD) and mitral valve dysplasia (MVD) have an incidence for CHDs of 3–3.5% and 1.5–2%, respectively. TVD is the most common CHD in Labrador retrievers, and it is transmitted via chromosome 9 by autosomal dominant with incomplete penetrance [21,44,45,46]. Heritability has also been established for Dogues de Bordeaux [47].

MVD is most commonly seen in large-breed dogs, and bull terriers and Great Danes are predisposed. In 36% of cases, the two defects occur together [48]. MVD and TVD include numerous malformations of the valvular apparatus, such as: shortening, rounding, the incomplete separation of the valve leaflets, short, thick or fusion of the chordae tendineae, direct insertion of the papillary muscle to the leaflet, and up malposition of the papillary muscle, causing malalignment of the chordae tendineae [44,45,46]. All these malformations can cause valve insufficiency or/and stenosis of a more or less severe degree [48]. Valvular insufficiency causes a volumetric overload of the atrium and ventricle of relevance, which leads to their enlargement over time. Puppies with MVD or TVD usually have a “plateau” murmur holosystolic or pansystolic type, with PMI at the focus of mitral or tricuspid auscultation, with an intensity directly proportional to the degree of severity of the regurgitation. In any case, a diastolic decrescendo murmur may be present. In particularly severe cases of TVD, the murmur may decrease or be completely absent, as the valve is so deformed that it does not generate any resistance to blood flow [48] (Table 2).

### 3.6. Tetrology of Fallot

Tetralogy of Fallot (TOF) is a rare defect, accounting for 1–3% of total CHDs in dogs [14,17]. In the Keeshonds breed, various genetic studies on TOF have been conducted, which indicate an onset of the defect linked to oligogenetic expression [49,50]. TOF is caused by abnormal embryonic development of the conotruncal septum, which deviates in an anterosuperior direction; this profoundly alters the geometry of cardiac development. In TOF, four main defects can be recognized:

- PS: With both infundibular and valvular components, often associated with pulmonary trunk hypoplasia.
- Concentric right ventricular hypertrophy: Secondary to the pressure overload due to PS.
- VSD: Generally large, it allows a right-left shunt due to the high resistance of the pulmonary circulation.
- Dextropositioned aorta [51].

The atrium and left ventricle are small, and non-oxygenated blood is introduced into the systemic circulation via the VSD. The degree of right-to-left shunting is influenced by the gravity of the PS and the systemic vascular resistance. Right-to-left shunting produces an arterial hypoxemia and an increase in blood viscosity, resulting in an activated erythropoietin production by the kidney [25,52].

The heart murmur is related to the degree of PS. In the presence of a mild PS and a small VSD, a loud right basilar holosystolic murmur caused by the VSD shunt is audible. Conversely, in the presence of a severe PS associated with a large VSD, and a hyperviscosity syndrome, the murmur may be inaudible [25,52] (Table 2).

**Table 2:** Breed predisposition and murmur description of most common canine CHDs.

Cardiac Heart Defect	Breed Predisposition	Murmur Decsription	References
Pulmonary Stenosis	bulldogs and boxers	Holosystolic, with rough timbre and PMI on the pulmonary valve focus, poorly or non-radiated, with a “crescendo-decrescendo” pattern	[13,14,15,16,17,18,19,20,21,22,23,24,25,26,27,28,29]
Patent ductus arteriosus	poodle, German shepherd dog, Pomeranian, Shetland sheep dog, collie, Maltese, Yorkshire terrier, chihuahua and Stabyhoun	Continuous (Gibson’s murmur), located in the left axillary region, with intensity ranged between IV/VI and VI/VI degrees.	[13,14,15,16,17,18,19,21,30,31,32,33,34,35,36,37,38]
Subaortic Stenosis	Large-breed dogs, such as the rottweiler, golden retriever and boxer	Holosystolic, “crescendo-decrescendo”, with a rough timbre, located at the focus of the aortic valve, can irradiate.	[13,14,15,16,17,18,19,20,21,22,23,24,25,26,27,28,29,39,40]
Aortic Stenosis	Large-breed dogs, such as the rottweiler, golden retriever and boxer	Holosystolic, “crescendo-decrescendo”, with a rough timbre, located at the focus of the aortic valve.	[13,14,15,16,17,18,19,20,21,22,23,24,25,26,27,28,29,39,40]
Ventricular Septal Defect	No breed predisposition has been reported	Holosystolic, II–III degree with PMI over the right heart base. A split S2 heart sound may be present.	[13,14,15,16,17,18,19,21,29,41,42,43]
Atrial Septal Defect	boxer, doberman, old English sheep dog and Samoyed	Holosystolic, II–III degree with PMI over the right heart base. A split S2 heart sound may be present.	[13,14,15,16,17,18,19,21,41,42,43]
Tricuspid valve dysplasia	Dogues de Bordeaux	“Plateau” murmur holosystolic or pansystolic, with PMI at the focus of tricuspid auscultation	[21,44,45,46,47,48]
Mitral valve dysplasia.	bull terrier and Great Dane	“Plateau” murmur holosystolic or pansystolic type, with PMI at the focus of mitral auscultation	[21,44,45,46,47,48]
Tetrology of Fallot	Keeshond	Depending to PS degree:loud right basilar holosystolic in presence of mild PS and a small VSD;absence in presence of severe PS and a large VSD.	[13,14,15,16,17,18,19,21,49,50,51,52]

All defects may be diagnosed in puppies almost 6–8 weeks of age.

## 4. Discussion

In puppies, various hereditary or congenital heart diseases are reported that can have important consequences on animal welfare and health. The diagnosis of CHD is challenging and time-consuming; often, CHDs are diagnosed beyond the legal warranty for vice protection [16]. In fact, often many CHDs have varying degrees of severity and development that for a long time may not give any symptoms other than a heart murmur. Some CHDs may also have minimal birth lesions but worsen over months, such as SAS, for example. Another key factor is that often, the veterinarian is not involved in the purchase but is consulted only after the onset of the first symptoms, which often occur beyond the first year of life [16].

The early identification of CHDs through murmur audibility can not only avoid legal disputes, but also improve prognosis and treatment. The diagnosis of cardiac murmurs can be challenging, but it represents a valid tool to help the veterinarian practitioner identify CHD early as possible, mostly in predisposed breeds.

Indeed, a puppy affected with a congenital defect may be considered unsuitable for its specified use. A cardiac defect, due to an anatomic alteration, causes a disorder in normal organic functioning, and inexorably determines a considerable impairment. CHDs may be qualified as a vice, in that the defect is:

1. Pre-existing or have a pre-existing cause;
2. Hidden (that is, it cannot be revealed during a routinary examination) or not easily recognized (at the time of purchase, it cannot be identified using the usual due diligence);
3. Serious or chronic: The defect, in relation its severity, renders the puppy unsuitable for the usage for which it is acquired, to such an extent that the buyer would not have bought it or would have given a lower price if he had been conscious of that.

Taking into account that in the sales process, animals are legally considered as personal property [53]—though they occupy an important role of a member of the household, much like children [54]—sale and purchase agreements afford buyers and vendors certain rights and responsibilities such as in the purchase of material goods [55,56,57].

In fact, in the EU, consumer protection concerning animal purchases is founded on the general consumer protection laws differently than in the US, where there are expressly pet purchase protection laws that vary in relation to the state. These laws allow from as few as 10 days (e.g., in Nevada for illness, disease, final state, or circumstance necessitating immediate hospitalization or surgery) to up to 2 years (e.g., in Rhode Island for congenital or hereditary disease) for the detection of the defect (period that is called time frame to exercise buyer remedies) and, necessitate that the clinical examination must be performed within a given number of days of purchase of the dog (usually within 15 business days) so that the buyer may exercise their own rights.

**Figure 2:**
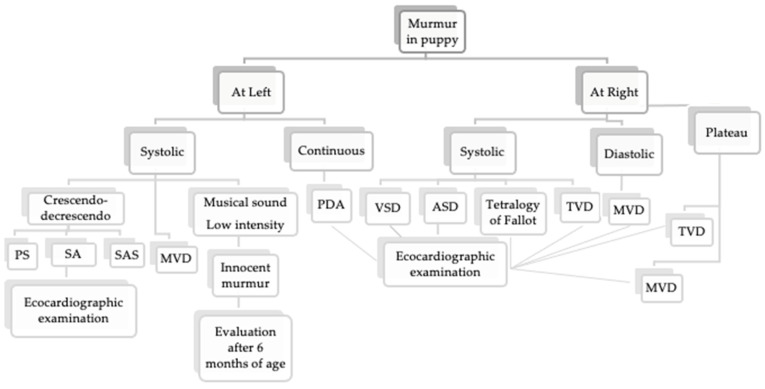
Protocol for assessment in presence of murmur in a >6-month-old puppy. PS, pulmonic stenosis; SA, stenosis aortic; SAS, stenosis subaortic; MVD, mitral valve dysplasia; TVD, tricuspid valve dysplasia.

An interesting question may be the occurrence that during the animal purchase, the buyer may consider the veterinarian responsible for failing to identify evident clinical signs of cardiac congenital/hereditary defects within the time provided from the law for identification in order to obtain required solutions. In these cases, it is important to follow rules of good practice and a systematic examination protocol, as here reported (Figure 2), that can prevent the adverse consequences of the possible defense lawsuit from dissatisfied buyers. In addition, there should be advisories to suggest the purchase of puppies from breeders who submit their breeding dogs to regular clinical and echocardiographic examinations and that are provided with CHD exemption certificates.

## 5. Conclusions

In conclusion, the veterinarian should be able to recognize and identify the murmur as in its severity degree and as a possible clinical sign of below congenital heart disease, with the aim of protecting not only the puppy’s welfare, but also the right of the owner.

## Figures and Tables

**Table 1:** Murmur grading scale in dogs.

Heart Murmur Grade	Description
GRADE 1	Very light murmur, difficult to hear, well localized.
GRADE 2	Light murmur but easy to recognize when the PMI is located.
GRADE 3	Modest murmur which has an intensity equivalent to normal heart sounds.
GRADE 4	Strong murmur but no palpable thrill.
GRADE 5	Strong murmur with palpable precordial thrill.
GRADE 6	Strong murmur with palpable thrill, even without the stethoscope.

## Data Availability

The data presented in this study are available on request from the corresponding author.
